# Open versus robotic retromuscular ventral hernia repair: outcomes of the ORREO prospective randomized controlled trial

**DOI:** 10.1007/s00464-024-11202-1

**Published:** 2024-09-12

**Authors:** Jeremy A. Warren, Dawn Blackhurst, Joseph A. Ewing, Alfredo M. Carbonell

**Affiliations:** 1 Prisma Health Department of Surgery, University of South Carolina School of Medicine Greenville, 701 Grove Rd, ST 3, Greenville, SC 29605 USA; 2 Prisma Health Research Administration, Greenville, SC USA; 3 Health Sciences Center, Prisma Health Upstate, Greenville, SC USA

**Keywords:** Robotic ventral hernia, Robotic retromuscular hernia repair, Open retromuscular hernia repair, Randomized controlled trial

## Abstract

**Background:**

Robotic retromuscular ventral hernia repair (rRMVHR) potentially combines the best features of open and minimally invasive VHR: myofascial release with abdominal wall reconstruction (AWR) with the lower wound morbidity of laparoscopic VHR. Proliferation of this technique has outpaced the data supporting this claim. We report 2-year outcomes of the first randomized controlled trial of oRMVHR vs rRMVHR.

**Methods:**

Single-center randomized control trial of open vs rRMVHR. 100 patients were randomized (50 open, 50 robotic). We included patients > 18 y/o with hernias 7–15 cm with at least one of the following: diabetes, chronic obstructive pulmonary disease (COPD), body mass index (BMI) ≥ 30, or current smokers. Primary outcome was occurrence of a composite outcome of surgical site infection (SSI), non-seroma surgical site occurrence (SSO), readmission, or hernia recurrence. Secondary outcomes were length of stay, any SSI or SSO, SSI/SSOPI, operative time, patient reported quality of life, and cost. Analysis was performed in an intention-to-treat fashion. Study was funded by a grant from Society of American Gastrointestinal and Endoscopic Surgeons.

**Results:**

90 patients were available for 30-day and 62 for 2-year analysis (rRMVHR = 46 and 32, oRMVHR = 44 and 30). Hernias in the open group were slightly larger (10 vs 8 cm, *p* = 0.024) and more likely to have prior mesh (36.4 vs 15.2%; *p* = 0.030), but were similar in length, prior hernia repairs, mesh use, and myofascial release. There was no difference in primary composite outcome between oRMVHR and rRMVHR (20.5 vs 19.6%, *p* = 1.000). Median length of stay was shorter for rRMVHR (1 vs 2 days; *p* < 0.001). All patients had significant improvement in quality of life at 1 and 2 years. Other secondary outcomes were similar.

**Conclusion:**

There is no difference in a composite outcome including SSI, SSOPI, readmission, and hernia recurrence between open and robotic RMVHR.

**Graphical abstract:**

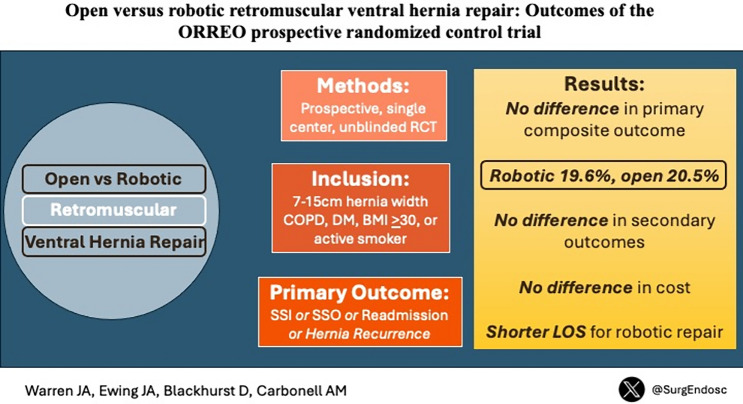

The exponential growth of robotic surgery in the hernia space is indicative of the innovative application of this technology. Enhanced 3-dimensional visualization, instrument dexterity, and surgeon ergonomics all contribute to the potential benefits to both patient and surgeon by enabling complex operations to be performed in a minimally invasive fashion. This is certainly the case in ventral hernia repair (VHR), where rectus abdominis release and transversus abdominis release (TAR) used for abdominal wall reconstruction (AWR), previously only performed using an open approach, have been adapted to the robotic platform. These techniques facilitate closure of the abdominal wall defect under lower tension and provide a wide extraperitoneal space for mesh reinforcement, but also carry significant wound morbidity. The ability, then, to perform a minimally invasive AWR that potentially reduces this risk is a significant advance. Indeed, early results of the safety and feasibility of robotic AWR are promising. A propensity-score matched analysis of the Abdominal Core Health Quality Collaborative (ACHQC) demonstrated similar clinical outcomes and a shorter length of stay after robotic retromuscular hernia repair (rRMVHR) [1]. A more recent study using similar methodology in patients at high risk for wound complications showed a reduction in surgical site infection (SSI) with rRMVHR compared to open RMVHR (4 vs 1%; *p* = 0.032) [2].

The interest and growth in robotic AWR have outpaced available high-quality data. As evidenced by the proliferation of retrospective series of robotic hernia repair, the growing membership in social media-based platforms focusing on robotic surgery and hernia repair, and review of any major general surgery society meeting schedule, there is great interest in robotic VHR. The Fellowship Council recently reported a dramatic increase in robotic hernia repair, increasing from 457 repairs in 2015 to 3391 in 2019, a volume increase of 266.3% per fellow [3]. Showing a similar trend, review of Medicare claims data demonstrated an increase in the proportion of ventral hernia repairs performed robotically from 2.1% in 2010 to 21.9% in 2020 [4]. However, much remains unknown about the operative risks, perioperative outcomes, optimal patient selection, and the ability to achieve both surgeon and patient goals for hernia repair. Intuitively, patients traditionally at high risk for wound complications might benefit most from a minimally invasive repair. Chronic obstructive pulmonary disease (COPD), diabetes mellitus (DM), smokers, and obese patients are generally considered at higher risk for wound complications and hernia recurrence [5–8] and were the target population for this study. We thus designed a prospective randomized trial to evaluate the impact of a robotic approach to AWR on outcomes in a group of high-risk patients. We hypothesized that robotic RMVHR would reduce occurrence of a composite clinical outcome compared with a traditional open retromuscular approach.

## Materials and methods

We performed a single center, unblinded, parallel randomized controlled trial comparing open retromuscular ventral hernia repair (oRMVHR) to robotic retromuscular ventral hernia repair (rRMVHR). The trial was conducted and reported according to the Consolidated Standards of Reporting Trials (CONSORT) guidelines [9]. The trial was registered with clinicaltrials.gov (NCT03007758) and approved by the Prisma Health Upstate Institutional Review Board. Data collection was completed using the Abdominal Core Health Quality Collaborative (ACHQC) registry. Additional data not contained in the ACHQC included randomization and cost data and was captured in the Research Electronic Data Capture (REDCap). There was one change in robotic hernia repair technique after study initiation. The initial robotic technique involved a transabdominal approach, most often including a bilateral TAR. Nine months after beginning the trial and with the initial 32 patients enrolled, our technique changed predominantly to an extended-view totally extraperitoneal (eTEP) approach. The result of this change was felt to be clinically minimal and nothing more than an alternative approach to access the retromuscular space rather than an altogheter different technique. In both variations, bilateral rectus abdominis release and retromuscular mesh placement is the same, with the only difference being an expected lower utilization of TAR for eTEP compared to the transabdominal approach.

Follow-up was planned for all patients at 30 days (range ± 14 days), 6 months (± 30 days), 1 year (± 45 days), and 2 years (± 60 days). Additional office visits were as needed but were not specifically tracked for study purposes unless related to any of the primary or secondary outcomes. Composite analysis was calculated based on the total number of randomized patients, and secondary outcomes were analyzed based on cumulative events rather than separated by time point. Follow-up included in-person visits, telehealth visits, review of the medical record, review of body imaging, or completion of PRO. Ten patients who were randomized were excluded from analysis due to failure to complete surgery (*n* = 7) or complete lack of follow-up (*n* = 3).

### Inclusion criteria

All patients ≥ 18 years old with a midline ventral or incisional hernia measuring 7–15 cm in widest dimension and at least one comorbidity associated with increased wound complications were eligible for enrollment. Qualifying comorbid conditions for inclusion were obesity, defined as a body mass index of ≥ 30 kg/m^2^, chronic obstructive pulmonary disease (COPD), diabetes mellitus (DM), or active smokers.

### Exclusion criteria

Patients were ineligible for participation if they were < 18 years old, had hernias < 7 cm or > 15 cm in widest dimension, had none of the above associated comorbid conditions, had a Center for Disease Control wound class 3 (contaminated) or 4 (dirty/infected), or presence of an enterostomy or parastomal hernia. There were three protocol deviations in which a parastomal hernia was present; two in the robotic group, and one open.

### Primary outcome

A composite outcome was designed based on common clinically relevant adverse outcomes of AWR. This was chosen for two reasons: First, an initial power analysis demonstrated an excessively high number of patients needed to demonstrate a benefit of rRMVHR over open repair for surgical site infection (SSI) or hernia recurrence alone and was not feasible. Second, while important, SSI is not the only perioperative complication that is relevant to patient recovery and quality of life; other surgical site occurrences (SSO), hospital readmission, and the need for reoperation or intervention for SSI or SSO can equally impact patient convalescence. The composite outcome included surgical site infection (SSI), surgical site occurrence (SSO; excluding simple seroma requiring no intervention), hernia-related hospital readmission, and confirmed hernia recurrence. Occurrence of one or more of these variables throughout the study period, regardless of complete follow-up, was considered a positive occurrence of the composite outcome.

### Secondary outcomes

Components of the composite outcome were analyzed individually, including SSI, SSO, and SSI or SSO requiring procedural intervention (SSO/SSIPI). Additional secondary outcomes were hospital length of stay, operative time, direct hospital cost, and patient-reported quality of life. Quality of life was measured using the Hernia-Related Quality of Life Survey [10] and pain score (0–10 visual analog scale). Hernia recurrence was assessed with a combination of clinical exam, radiographic exam, and/or Ventral Hernia Recurrence Inventory [11]. Composite hernia recurrence includes any patients with objectively confirmed hernia recurrence by physical exam or imaging, patients reporting bulging or recurrence by VHRI, and excludes patients who report bulging or recurrence by VHRI who have negative objective findings on exam or imaging for hernia recurrence.

### Power analysis

Target study enrollment was determined using internal retrospective data. Analysis of a cohort of rRMVHR and oRMVHR from our institution that met the above inclusion and exclusion criteria demonstrated occurrence of the primary outcome in 24.1 and 52.2%, respectively. Using a power of 80% and significance of 5%, we estimated that 46 patients in each group were needed to demonstrate a significant difference in the primary endpoint. To account for patient withdrawal and failure to maintain follow-up, we aimed to enroll 50 patients in each arm.

### Randomization

Randomization was conducted using a simple allocation method in a 1:1 ratio using REDCap randomization module. Neither patients nor surgeons were blinded to the intervention or outcomes. Randomization occurred during the preoperative encounter after research consent was obtained.

### Statistical analysis

Analysis was performed in an intention-to-treat manner. Bivariate analysis comparing rRMVHR and oRMVHR were conducting using Fisher’s Exact test for categorical data and the Wilcoxon Rank Sum text for continuously distributed date. The Wilcoxon Signed Rank test was used to test for within-group differences between baseline and follow-up pain scores and HerQLes scores. All statistical analyses were completed using SAS statistical software (SAS Enterprise Gide 8.3, Cary, NC). *P* values of < 0.005 were considered indicative of statistical significance.

## Results

**Fig. 1:**
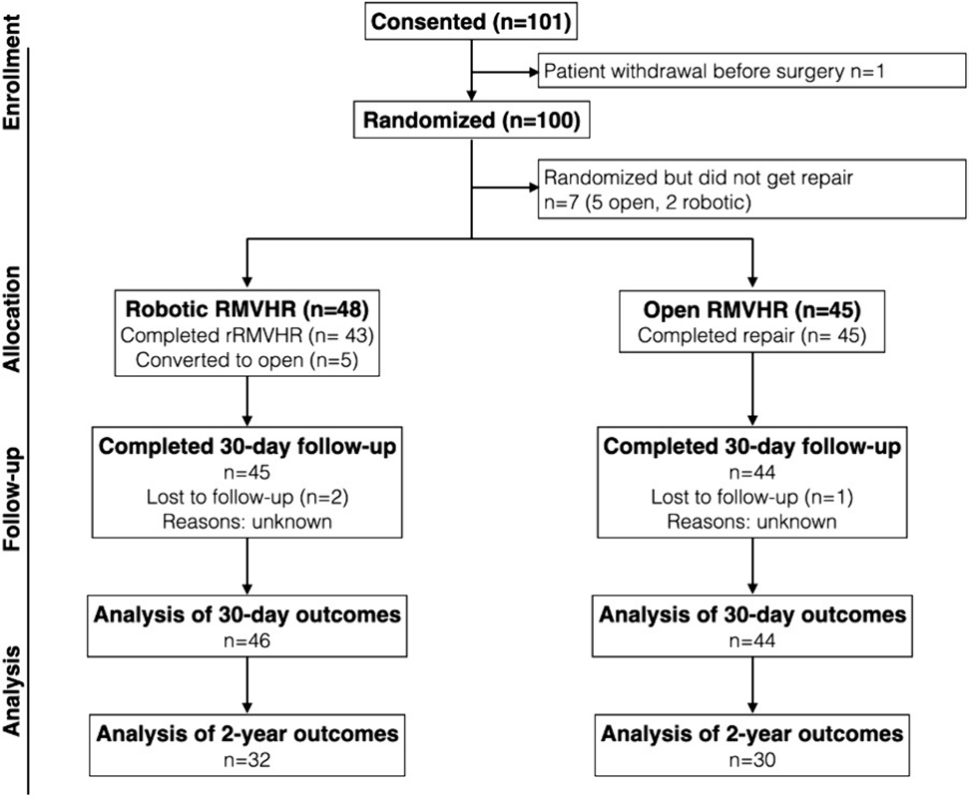
CONSORT flow diagram

After screening, 101 patients were consented. One patient withdrew from the study prior to surgery, leaving 100 patients randomized for inclusion. A total of 7 patients were randomized but never went through with surgery (5 randomized to open, 2 randomized to robotic repair). Two patients in the robotic arm and one in the open arm did not complete 30-day follow-up and were excluded, leaving 46 rRMVHR and 44 RMVHR patients for analysis of the primary outcome. Five robotic cases required conversion to open and remained in the rRMVR group for intention-to-treat analysis. Four patients died during the study period (two rRMVHR, two oRMVHR) of unrelated causes, and 24 patients were lost to follow-up, leaving 62 patients (32 rRMVHR and 30 oRMVHR) completing 2-year follow-up (Fig. 1). There were 3 protocol deviations in the open group, all of which were repaired with extraperitoneal mesh in the preperitoneal rather than retromuscular position. These were included in the study and analysis.

There were no differences between groups in age, gender, BMI, comorbidity, or insurance status (Table 1). Regarding variables analyzed for the composite outcome, the median BMI was 36.7 for both groups (*p* = 0.774), diabetes present in 28.3% of rRMVHR and 36.4% of oRMVHR (*p* = 0.500), COPD in 15.2 and 13.6%, and active smokers in 34.8 and 31.8%. Hernia characteristics and repair techniques were similar between groups, with the exception of slightly larger hernias in the open group and a higher incidence of prior mesh in the open group. The median hernia width for RMVHR was 10 cm compared to 8 cm for rRMVHR (*p* = 0.024). Prior mesh was present in 36.4% of open cases compared to just 15.2% of robotic cases (*p* = 0.030). Transversus abdominis release was required in 30.4% of rRMVHR and 22.7% of oRMVHR (*p* = 0.205) (Table 1).

**Table 1:** Study population details: patient characteristics, hernia characteristics, and operative details

Characteristic	Robotic	Open	*p* value
Patient details			
N	46	44	–
Age (years)			
Median (IQR)	59.5 (50, 67)	59.5 (47, 66)	0.686
Min, Max	28, 76	32, 75	
Gender: *n* (%)			
Female	28 (60.9)	30 (68.2)	0.514
Male	18 (39.1)	14 (31.8)	
Comorbidities: * n* (%)			
HTN	26 (56.5)	28 (63.6)	0.525
DM	13 (28.3)	16 (36.4)	0.500
COPD	7 (15.2)	6 (13.6)	1.000
Active Smoker	16 (34.8)	14 (31.8)	0.825
BMI: Median (IQR)	36.7 (30.6, 41.9)	36.7 (31.6, 40.2)	0.774
ASA: * n* (%)			
2	13 (28.3)	12 (27.3)	1.000
3	32 (69.6)	32 (72.7)	
4	1 (2.2)	0 (0.0)	
Hernia/operative details			
Hernia type: * n* (%)			
Incisional	44 (95.7)	41 (93.2)	0.421
Primary ventral	2 (4.3)	1 (2.3)	
Parastomal	0 (0.0)	2 (4.5)	
Recurrent hernia? * n* (%)	16 (34.8)	23 (52.3)	0.136
Number of prior repairs			
None	30 (65.2)	21 (47.7)	0.066
1	10 (21.7)	20 (45.5)	
2	5 (10.9)	2 (4.5)	
3 or more	1 (2.2)	1 (2.3)	
Hernia width: median (IQR)	8 (6, 10)	10 (8, 12)	0.024*
Hernia length: median (IQR)	15 (12, 20)	15 (10, 19)	0.363
Prior mesh present: * n* (%)	7 (15.2)	16 (36.4)	0.030*
Myofascial release: * n* (%)			
Posterior rectus sheath only	32 (69.6)		0.205
TAR	14 (30.4)	10 (22.7)	
Prior mesh removed? * n* (%)			
Intraoperative complications: * n* (%)	6 (13.0)	4 (9.1)	0.740
Bowel injury (serosa)	6 (100)	2 (50.0)	
Bowel injury (full-thickness)	0 (0.0)	2 (50.0)	
Conversion to open	5 (10.9)	n/a	

Statistical tests applied: Wilcoxon Rank Sum for age, hernia width, hernia length; Fisher’s Exact text for all other variables

*IQR* interquartile range, *HTN* hypertension, *DM* diabetes mellitus, *COPD* chronic obstructive pulmonary disease, *BMI* body mass index, *ASA* American Society of Anesthesia score, *TAR* transversus abdominis release

*Indicates statistically significant result

No difference was seen in the primary composite outcome between groups, occurring in 9 (19.6%) of rRMVHR and 9 (20.5%) oRMVHR cases (*p* = 1.000) (Table 2). Analysis of secondary outcomes demonstrated no difference between rRMVHR and oRMVHR in the rate of simple seroma (17.4 vs 15.9%, *p* = 1.000), SSI (4.4 vs 6.8%; *p* = 0.673), readmission (6.5 vs 2.3%; *p* = 0.617), hernia recurrence (2.2 vs 0%; *p* = 0.001), total SSO (47.8 vs 29.6%; *p* = 1.000), or SSOPI (13 vs 6.8%; *p* = 0.486). Median length of stay (LOS) was significantly shorter after rRMVHR compared to open (median 1 vs 2 days; *p* < 0.001). Operative time was significantly longer with rRMVHR (*p* = 0.003) (median 206 vs 156 min; *p* < 0.001). Direct and total hospital cost were not statistically different between rRMVHR and oRMVHR (median total cost $11,747 vs $9267; *p* = 0.092; median direct cost $6084 vs $4826; *p* = 0.317). Table 3 details secondary outcome analysis.

**Table 2:** Analysis of primary and secondary outcomes

Outcome	Robotic	Open	*p* value
*n* included in analysis	46	44	
Primary composite outcome: * n* (%)	9 (19.6)	9 (20.5)	1.000
SSO (excluding simple seroma)	8 (17.4)	7 (15.9)	1.000
SSI	2 (4.4)	3 (6.8)	0.673
Hernia-related readmission	3 (6.5)	1 (2.3)	0.617
Hernia recurrence (confirmed)	1 (2.2)	0 (0.0)	1.000
Secondary outcomes: * n* (%)			
Any SSO/SSI	22 (47.8)	13 (29.6)	1.000
SSO/SSI PI	6 (13.0)	3 (6.8)	0.486
Reoperation	0 (0.0)	1 (2.3)	0.489
LOS (days)			
Median (IQR)	1 (0, 2)	2 (1, 3)	< 0.001*
Min, Max	0, 5	0, 5	
Operative time range (minutes)			
60–119	3 (6.5)	6 (13.6)	0.003*
120–179	11 (23.9)	24 (54.5)	
180–239	25 (54.4)	9 (20.5)	
≥ 240	7 (15.2)	5 (11.9)	
Operative time			
Median (IQR)	206 (184, 237)	156 (130, 205)	< 0.001*
Mean (± SD)	219 ± 73.5	171.9 ± 63.6	< 0.001*
Cost: U.S. Dollars ($), Median (IQR)			
Total cost	11,747 (9717, 13,530)	9267 (8873, 11,641)	0.092
Direct cost	6084 (4799, 6878)	4826 (4450, 5966)	0.137

Statistical tests applied: Wilcoxon Rank Sum for LOS, operative time (in minutes), and cost. Fisher’s Exact test for the remaining variables, including categorical operative time

*SSO* surgical site occurrence, *SSI* surgical site infection, *SSO/SSIPI* SSO or SSI requiring procedural intervention, *LOS* length of stay, *IQR* interquartile range, *SD* standard deviation

*Indicates statistically significant result

**Table 3:** Patient-reported outcomes

	Robotic	Open	*p* value
HerQLes: median (IQR)			
30-days: *n*	41	41	–
Baseline PRO	34 (16, 52)	28 (16, 64)	0.628
30-day PRO	38 (18, 68)	50 (34, 58)	0.325
Difference	0 (-22, 32)	16 (-20, 30)	0.339
*p* value (compared to baseline)	0.641	0.119	–
6–12 months: *n*	39	33	–
Baseline PRO	26 (12, 40)	46 (34, 80)	< 0.001*
6 or 12-month PRO	86 (64, 94)	90 (72, 96)	0.590
Difference	60 (22, 78)	20 (10, 54)	0.008*
*p* value (compared to baseline)	< 0.001*	< 0.001*	–
24 months: *n*	32	30	–
Baseline PRO	36 (21, 69)	32 (16, 50)	0.359
24-month PRO	80 (42, 92)	91 (76, 94)	0.165
Difference	28 (-5, 56)	59 (14, 64)	0.016*
*p* value (compared to baseline	0.002*	< 0.001*	–
Pain score: median (IQR)			
Baseline PRO	2 (1, 2)	3 (2, 3)	0.027*
30-day PRO	3 (2, 3)	2 (2, 3)	0.020*
Difference	1 (0, 2)	0 (-1, 1)	0.006*
*p* value (compared to baseline)	0.005*	0.404	–
Baseline PRO	2 (2, 3)	2 (1, 3)	0.020*
6 or 12-month PRO	1 (1, 2)	1 (1, 2)	0.805
Difference	-1 (-2, 0)	-1 (-1, 0)	0.170
*p* value (compared to baseline)	< 0.001*	0.078	–
Baseline PRO	2 (2, 3)	2 (2, 3)	0.123
24-month PRO	1 (1, 2)	1 (1, 1)	0.286
Difference	-1 (-1.5, 0)	-1 (-2, 0)	0128
*p* value (compared to baseline)	0.027*	< 0.001*	–
Ventral Hernia Recurrence Inventory: *n*/total (%)			
Pain at hernia site?			
1 year	9/39 (23.1)	5/33 (15.2)	0.552
2 years	8/32 (25)	4/30 (13.3)	0.339
Bulging at hernia site?			
1 year	16/39 (41)	7/33 (21.2)	0.083
2 years	11/32 (34.3)	3/30 (10%)	0.033*
Do you feel your hernia has come back?			
1 year	2/39 (5.1)	2/33 (6.1)	1.000
2 years	7/32 (21.9)	1/30 (3.3)	0.054
VHRI recurrence at 2 years	11/32 (34.4)	4/30 (13.3)	0.076
Composite recurrence	4 (12.5)	1 (3.3)	0.355

Statistical tests applied: Wilcoxon Rank Sum for between-group differences and within-group differences in HerQLes and pain scores; Fisher’s Exact test for VHRI

*HerQLes* Hernia related quality of life score, *IQR* interquartile range, *PRO* patient reported outcome, *VHRI* ventral hernia recurrence inventory

*Indicates statistically significant result

Patient reported outcomes (PRO) were collected at baseline prior to surgery, and at 30-day, 6-month, 12-month, and 24-month follow-up (Table 3). Data for 6 and 12-month PRO were combined for analysis. Patients reported similar HerQLes scores at baseline (*p* = 0.628) and no differences between groups at 30-days (*p* = 0.325), at 6–12 months (*p* = 0.590), or at 24-months (*p* = 0.165). The relative improvement above baseline was greater for rRMVHR at 6–12 months (*p* = 0.008) and greater for oRMVHR at 24-months (*p* = 0.016). Overall, both groups showed significant improvement at 6–12 and 24 months over baseline, however. Pain scores were lower for oRMVHR at 30-day follow-up (median 2 vs 3; *p* = 0.020), similar at 6–12 months (median 1 vs 1; *p* = 0.805), and similar at 24 months (median 1 vs 1; *p* = 0.286). Overall, both groups had lower pain scores at 6–12 and 24-month follow-up compared to baseline. Hernia recurrence was assessed by clinical exam, computed tomography (CT), and VHRI. For VHRI at 1-year (*n* = 39 rRMVHR, *n* = 33 oRMVHR), patients reported pain at the hernia site in 9 (23.1%) rRMVHR and 5 (15.2%) oRMVHR (*p* = 0.552), bulging at the hernia site in 16 (41%) rRMVHR and 7 (21.2%) oRMVHR (*p* = 0.083), and the feeling that the hernia recurred in 2 (5.1%) rRMVHR and 2 (6.1%) oRMVHR (*p* = 1.00). At 2 years (*n* = 32 and 30, respectively), pain at was reported in 8 (25%) rRMVHR and 4 (13.3%) oRMVHR (*p* = 0.339), bulging in 11 (34.3%) rRMVHR and 3 (10%) oRMVHR (*p* = 0.033), and feeling of recurrence in 7 (21.9%) rRMVHR and 1 (3.3%) oRMVHR (*p* = 0.054). Cumulative possible hernia recurrence based on VHRI alone was 11 (34.4%) for rRMVHR and 4 (13.3%) for oRMVHR (*p* = 0.076). For oRMVHR, 3 of the 4 possible hernia recurrences reported by VHRI had CT imaging (*n* = 2) or physical exam (*n* = 1) excluding hernia recurrence, leaving 1 possible hernia recurrence (3.3%). For 11 patients reporting possible recurrence after rRMVHR, 1 patient developed a separate trocar site hernia, 1 developed a separate incisional hernia at a prior colostomy site, 5 had imaging excluding hernia recurrence, and 1 had a recurrent hernia that was repaired, leaving 3 (9.4%) additional possible hernia recurrence at 2 years based on VHRI. Thus, total composite recurrence for open vs robotic RMVHR was 3.3 and 12.5% (*p* = 0.355), respectively. PROs are detailed in Table 3.

## Discussion

Based on the results of this randomized controlled trial, postoperative outcomes are similar after both open and robotic RMVHR. This finding holds true in analysis of the primary composite outcome as well as secondary outcomes, with the exception of a shorter length of stay after robotic repair. This is in contradistinction to the lower wound morbidity associated with laparoscopic VHR [12–14]. Our results are consistent with the existing literature on rRMVHR, which has yet to clearly demonstrate a benefit in wound morbidity. Early retrospective reports by Bittner, et al. and Martin-del-Campo, et al. comparing oRMVHR and rRMVHR failed to demonstrate a significant difference in wound morbidity [15, 16]. A recent study by Kudsi, et al. did demonstrate a benefit of robotic VHR over open VHR (7.3 vs 2.5%; *p* < 0.001; unweighted analysis), but there was significant heterogeneity in this study, with only 8% of open repairs and 49.8% of robotic repairs performed in a retromuscular fashion [17]. Initial analysis from the first prospective observational study to compare open, robotic, and laparoscopic VHR, LeBlanc, et al. report a similar rate of post-operative complications across all repair types. There was no standardization of nor reporting of the specific techniques used for hernia repair in this study, however [18]. The most recent comparative analysis of open vs robotic RMVHR by Gaskins, et al., analyzed a propensity score matched cohort of patients from the ACHQC. This is the first study to demonstrate a statistically significant lower rate of SSI for rRMVHR (4 vs 1%; *p* = 0.032) and lower rate of SSOPI (9 vs 3%; *p* = 0.015) [2]. This study does report a lower incidence of both SSI and SSO compared to this study. This is likely due in part to selection bias of the retrospective sample analyzed and reporting bias, as data into the ACHQC is voluntary and surgeon entered.

The single clear demonstrable difference in all these studies, as well as the current trial, is a shorter LOS for robotic repair than open. Hospital LOS was 1 day shorter in this study for rRMVHR. Reasons for this difference are not clear. Anecdotally, we expected the difference might result from less pain after robotic repair. However, reported pain and quality of life at initial follow-up demonstrated slightly higher pain scores after rRMVHR compared to oRMVHR, with similar HerQLes scores. Future study to look more closely at in-hospital and earlier pain and quality of life measures, including opioid use, may yet elucidate the cause for this difference. Long-term, all patients reported improvement in quality of life after repair in both groups, with no significant differences between rRMVHR and oRMVHR. We did find that more patients reported bulging at 1 year after rRMVHR compared to open (34.3 vs 10%; *p* = 0.033), a trend that continued through 2-year follow-up. The reason for this is unclear. Ultimately, there was no statistical difference in composite hernia recurrence (3.3% oRMVHR vs 12.5% rRMVHR), but this finding is concerning, and additional long-term follow-up is needed.

While this study does not demonstrate superiority of rRMVHR over open, neither does it show inferiority. Robotic assisted surgery remains a valuable tool in the armamentarium of hernia repair techniques, and future studies may yet identify significant differences in outcomes. Patient selection is complex and the inclusion criteria for this trial may not be representative of the ideal rRMVHR patient. Cosmesis, prior surgical history, and perception of surgical approach can all influence the shared-decision making during preoperative assessment and were not fully accounted for. As such, this trial should be considered confirmation that a rRMVHR is a safe and feasible option with at least comparable results to traditional open repair. Of concern is the trend toward higher recurrence, particularly using the VHRI. Since there is not yet any long-term data on the durability of rRMVHR, this should be considered and discussed with patients. Theoretically, recurrence should be comparable to open repair as the end result—retrorectus dissection ± TAR, layered closure of the posterior and anterior sheath, and retrorectus mesh placement—is essentially identical. Further study with long-term follow-up is needed to determine the risk of recurrence.

As noted above, this study used a composite outcome as our primary endpoint. While not a novel concept, this outcome is not commonplace in surgical literature. The use of composite outcomes is particularly useful when there are potentially multiple outcomes relevant to the treatment. In our case, SSI alone is important, but other wound complications, hospital readmission, and hernia recurrence are arguably equally important clinical outcomes. The use of the composite outcome also increases the statistical precision of a trial, thus enabling greater power with a smaller sample size [19]. In our initial planning of this trial, power analysis calculations to demonstrate statistical differences in any single variable of the composite outcome resulted in prohibitively high sample size needed for enrollment. Indeed, even using this methodology, patient recruitment proved difficult, and the study period was significantly prolonged beyond initial expectations.

This is the first prospective randomized controlled trial comparing open and robotic retromuscular ventral hernia repair and a significant step forward in quantifying the impact of robotic surgery on VHR. However, there are several limitations that should be addressed. First, though we attempted to identify patients at highest risk for wound morbidity, hypothesizing this is where the greatest difference would be found, this may in fact not be the population with the greatest benefit of robotic repair. Other significant factors that may impact outcomes, such as cosmetic appearance, early postoperative pain, opioid use, and return to work or other activity, were not captured in this study. Patient expectations and their desired outcome can greatly influence their preferred approach and impact their satisfaction and postoperative quality of life following VHR. Indeed, we found this to be a significant factor in recruitment, with a much greater than anticipated number of patients unwilling to be randomized because they preferred an open or robotic approach specifically. Another important limitation was follow-up. We ultimately had complete follow-up of only 62% of enrolled patients and 69% of patients who had their hernia repaired. This effectively resulted in an underpowered study to detect the hypothesized difference in the primary outcome. However, extending the trial for up to an additional 2 years to improve recruitment and follow-up was not logistically feasible. Despite this fact, we believe this study does demonstrate equipoise of open and robotic RMVHR and justifies future study with larger sample size and robust follow-up.

## Conclusion

In the first randomized controlled trial comparing open and robotic retromuscular ventral hernia repair, there is no difference in a composite outcome including SSI, SSO, readmission, and hernia recurrence. Though this trial does not establish superiority, it does confirm the safety and feasibility of rRMVHR, and it should be considered a comparable repair technique to traditional open retromuscular repair.
